# *What’s love got to do with it?* Adolescent romantic networks and substance use

**DOI:** 10.1080/02673843.2015.1122643

**Published:** 2015-12-24

**Authors:** Wura Jacobs, Kwon Chan Jeon, Patricia Goodson, Thomas W. Valente

**Affiliations:** a Department of Health and Kinesiology, Texas A&M University, College Station, TX, USA; b Department of Health and Human Performance, Northwestern State University, Natchitoches, Louisiana, USA; c Department of Preventive Medicine, University of Southern California, Los Angeles, California, USA

**Keywords:** adolescents, romantic networks, substance use, romantic aspirations/relations, smoking, alcohol use

## Abstract

This study examined how romantic aspiration network characteristics at the individual level (in-degree and out-degree) are associated with substance use (i.e. smoking and drinking) among a cross-sectional sample of US adolescents (10th grade, *n* = 1523) from 4 high schools in Los Angeles. Findings highlighted that, with an increase in out-degree (romantic aspiration nominations made), adolescents in our sample were less likely (OR = .824, CI = .688–.986, *p* < .05) to report smoking in the past 30 days. Additionally, with an increase in in-degree (romantic aspiration nominations received), adolescents were more likely (OR = 1.186, CI = 1.04–1.36, *p* < .05) to report drinking in the past 30 days. We conclude that romantic aspirations/relations influence adolescents’ substance use behaviour (i.e. smoking and drinking alcohol), particularly because of the intensity of such relationships and the desire to please or be acceptable to the other person. Moreover, understanding adolescents’ aspirations/relations can be useful for the development of intervention/prevention programmes to target adolescents’ substance use.

## Introduction

Until recently, social scientists paid limited attention to adolescent romantic relationships because these relationships were viewed as fleeting and superficial (Collins, 2003; Smetana, Campione-Barr, & Metzger, 2006). However, research on adolescents’ romantic ties is burgeoning with studies showing that by 10th grade, interactions with romantic partners are much more frequent than interactions with parents, siblings or peers. Additionally, researchers are finding that adolescents who begin dating early have poorer psychosocial adjustment, including lower self-esteem, lower academic achievement, more alcohol and substance abuse and earlier involvement in sexual activity (Bouchey & Furman, 2006; Collins, 2003; Engels & Knibbe, 2000; Laursen & Williams, 1997; Smetana et al., 2006). Hence, it is crucial to understand the impact of romantic relationships on adolescents’ social development, health and well-being, particularly their influence on substance use behaviours.

Before romantic relationships are formed, they usually begin as an aspiration or attraction or a ‘crush’ and studies have shown that adolescents spend a lot of time thinking about these romantic attractions/potential relationships (Smetana et al., 2006). During this stage of attraction, many teens identify mutual interests and contexts which can serve as meeting opportunities. These mutual interests include activities such as parties, sports, clubs, engaging in deviant behaviours or using substances such as alcohol and tobacco (Engels & Knibbe, 2000; Engels & ter Bogt, 2001). Thus, it is important to study existing romantic relationships/couplings and how the stage of romantic aspiration/attraction influences adolescent substance use behaviour.

Smetana et al. (2006) stated that romantic relationships are integral and crucial to adolescents’ psychosocial development, social esteem and self-worth. Formation of romantic relationships is a significant developmental task of adolescence and these relationships have been shown to significantly influence adolescents’ health and lifestyle choices (Furman & Shaffer, 2003). Consequently, teenagers will do many things to become romantically desired by peers. Some adolescents go as far as changing their appearance to match the preferences of the other peer to whom they are romantically attracted, or engage in false self-behaviour (acting in ways that are not their true selves) in order to seem attractive (Steinberg & Morris, 2001).

Given that adolescents value romantic relationships as a means of establishing their own identities apart from their parents, multiple, simultaneous determinants such as (1) individual factors (hormonal changes during adolescence, heightened allure of experiencing intimate relationships and academic performance); (2) intra-family influence (parent and sibling substance use habits); and (3) extra-family influence (peer and romantic connections) may increase adolescents’ susceptibility to engage in substance use behaviour as a means to cope or as a means to become socially acceptable enough to find or be a date (Engels & Knibbe, 2000; Steinberg & Morris, 2001).

Adolescents, by reason of their developmental stage and age, are at a higher risk for engaging in substance use. According to the national Youth Risk Behavior Survey (YRBS) by the Centers for Disease Control and Prevention (CDC), 41.1% of adolescents have reported trying cigarette smoking and 66.2%reported having had at least one alcoholic drink (Centers for Disease Control & Prevention, 2013). In many cases, early involvement with alcohol and smoking, initiated during adolescence, is associated with risky behaviours such as delinquency, crime and sexually risky practices (Jackson, Geddes, Haw, & Frank, 2012). Moreover, engaging in these behaviours can lead to significant acute or chronic health outcomes and social and economic problems for these adolescents as adults, for their families and for society at large (Hingson, Heeren, Winter, & Wechsler, 2005; Kreager, Haynie, & Hopfer, 2013; Mundt, 2011; Osilla et al., 2014; Sitnick, Shaw, & Hyde, 2014).

Researchers have identified peer relationships among adolescents (e.g. romantic ties and/or friendships) as key factors that encourage and support alcohol or smoking initiation among adolescents (Alexander, Piazza, Mekos, & Valente, 2001; Fujimoto & Valente, 2012; Kreager & Haynie, 2011; Jeon & Goodson, 2015). Hence, to better understand how romantic ties and friendship relationships can influence adolescents’ substance use, social network analysis (SNA) represents an optimal approach. SNA comprises a set of theories and methods that employ a relationally based approach for studying the content and context of peer associations (Valente, 2015, 2010). As such, SNA is considered an appropriate method specifically for studying peer influences on adolescent substance use (Ennett et al., 2006; Valente, Gallaher, & Mouttapa, 2004).

One interesting feature of SNA is how it allows researchers to examine adolescent networks in terms of the adolescent’s position within the net of relationships. Two of the most widely used measures of network position are *in*-*degree* and *out*-*degree*. In-degree (or the number of nominations a particular adolescent receives) assesses an adolescent’s ‘popularity’ or prestige; out-degree (or the number of outgoing nominations) assesses an adolescent’s ‘gregariousness’ or sociability (Borgatti, Everett, & Johnson, 2013).

We, therefore, measured adolescents’ romantic desirability (i.e. in-degree: number of romantic nominations received) or desire to date (i.e. out-degree: number of outgoing romantic nominations) and their association with adolescents’ 30-day alcohol or tobacco use. Succinctly stated, the aim of this study was to examine a sample of adolescents’ romantic aspiration networks and their associations with alcohol and tobacco use.

## Methods

Data for this study are part of the pilot for The University of Southern California Social Networks Study. The district superintendent, principal and teachers in each of the included schools gave permission to conduct the study. Approval to conduct the survey also was obtained from the University of Southern California’s Institutional Review Board. Cross-sectional samples of US adolescents (10th grade) from 4 high schools in a school district in Los Angeles completed a paper and pencil survey in May 2010. Approximately, 1224 students returned their parental consent forms and 1,110 students completed the surveys on a regular school day during either an English or History class. Overall participation rate was 73%.

### Outcome variables

Alcohol use was assessed via the item ‘During the past 30 days, on how many days did you have at least one drink of alcohol?’ Response options comprised 0 days; 1 or 2 days; 3–5 days; 6–9 days; 10–19 days; 20–29 days; and all 30 days. Given that majority of the respondents (64.5%) reported never consuming alcohol, responses were dichotomised into two groups: ever drinkers or never drinkers.

Similarly, tobacco use was assessed via the item ‘During the past 30 days, on how many days did you smoke cigarettes?’ Response options were 0 days; 1 or 2 days; 3–5 days; 6–9 days; 10–19 days; 20–29 days; and all 30 days. Again, given that the majority of respondents (90.5%) reported never smoking cigarettes, responses were dichotomised into ever smokers or never smokers.

### Romantic aspiration networks

Students’ romantic aspiration networks were mapped by providing a roster of all 10th graders with an accompanying photo of each student and an ID number (created for each student by the researchers) printed on the bottom of the pictures. Students were asked to list five students in the grade with whom they *would like* to have a romantic relationship. The networks elicited by this question will be referred to, in this report, as ‘romantic networks’.

The network boundary for this investigation was intentionally set to the grade level for two reasons. First, a study published from similar data showed there was no difference in the magnitude of associations when the boundary was set at either the classroom or the grade level (Valente, Fujimoto, Unger, Soto, & Meeker, 2013). Second, extending the boundary beyond the classroom provides the opportunity to capture a wider range of peer interactions.

### Network measures

Two network measures were calculated: in-degree – the number of directional links to an ego (student) from other actors (other students), also known as incoming nominations (Valente, 2010), and out-degree – the number of directional links from an actor to other actors (outgoing nominations) (Valente, 2010). All network analyses were conducted using UCINET 6.5 (Borgatti, Everett, & Freeman, 2002).

### Data analysis

Logistic regression models were calculated to examine the association between adolescents’ romantic network characteristics and their smoking and drinking status (whether a student consumed tobacco or alcohol in the past 30 days), while controlling for several covariates known to influence the frequency and quantity of alcohol consumption and cigarette smoking (Marschall-Lévesque, Castellanos-Ryan, Vitaro, & Séguin, 2014). For our purposes, covariates included: sibling and parent alcohol/tobacco consumption, age, sex, race (Hispanic or Non-Hispanic), academic achievement (coded into four categories as shown in Table 1) and qualifying for reduced lunch (Yes or No). Study participants were recruited from four schools (similar in terms of demographics), however, to control for variances in other school characteristics; schools were dummy coded and included in the model with School 4 designated as the reference group. All logistic regression analyses were conducted in SPSS 23.

## Results

### Descriptive statistics

Table 1 details the demographic characteristics of study participants across the four schools. Study respondents were evenly distributed across gender and were on average 15.6 (SD = .584) years old. Consistent with the ethnic distribution in the area in which the data were collected, the students in the study were predominantly Hispanic/Latino. Socio-economic status was represented by whether students qualified for reduced lunch.

While the schools varied with regard to their 10th-grade students’ alcohol use, overall, 35.5% of the total sample reported consuming alcohol within the past month, not including those who drank for religious purposes. More than a third of the students surveyed (35.1%) also reported having a sibling who consumed alcohol. Parental alcohol use varied across the schools (from 45 to 57%), but for the total sample, more than half (52.1%) reported having a parent who drank alcohol. Most students (90.5%) reported not having ever smoked and 16.6% reported having siblings who smoked. Parental tobacco use varied across the schools but 28.2% of all the students reported having a parent who smoked.

### Network outcomes

Regarding network characteristics, mean out-degree and in-degree scores are shown in Table 1. School 4 had the lowest mean out-degree scores (nominations made), .76 (SD = 1.51), while School 3 had the highest out-degree average (with high variability) among all four schools, 1.27 (SD = 1.72). Logistic regression analyses were performed to ascertain the effects of intrapersonal factors (age, gender, ethnicity and academic achievement), interpersonal factors (qualifying for reduced lunch, parental and sibling alcohol use and smoking) and romantic network characteristics (in-degree and out-degree) on the likelihood that participants drank or smoked cigarettes in the past 30 days.

For the model with smoking as the outcome variable (Table 2), the logistic regression model was statistically significant (*χ*^2^ (10) = 75.76; *p* < .000). The model explained 17.8% (Nagelkerke *R*^2^) of the variance in adolescents’ 30-day tobacco use and correctly classified 91.7% of the teens in our sample. With increases in the number of romantic aspiration nominations (out-degree), adolescents in our sample were less likely to report smoking in the past 30 days (AOR = .824, CI = .688–.986; *p* < .05).

There was no association between nominations received (in-degree) and smoking in our sample. However, adolescents who reported having sibling(s) who smoked were nearly four times more likely (OR = 3.80, CI = 2.215–6.51; *p* < .0001) to report smoking in the past 30 days compared to those who did not have siblings who smoked. Age (OR = 2.11; CI = 1.361–3.28; *p* < .001) and decreasing academic achievement also were associated with an increased likelihood of smoking, but qualifying for reduced lunch was not associated with smoking.

Similar to smoking, the regression model with alcohol use as outcome variable (shown in Table 3) was statistically significant (*χ*^2^ (10) = 152.98; *p* < .0001). The model explained 21.1% (Nagelkerke *R*^2^) of the variance in adolescents’ 30-day alcohol use and correctly classified 70.6% of the teens in our sample. Unlike results obtained in the model for smoking, however, in-degree (and not out-degree) was associated with drinking. With every unit increase in in-degree (i.e. more romantic nominations received), adolescents in our sample were slightly more likely to report drinking in the past 30 days (OR = 1.186; CI = 1.04–1.36; *p* < .05).

Out-degree was not associated with alcohol use in our sample. Both parental and sibling alcohol use were associated with adolescents’ alcohol use. Compared to those who reported having a parent or sibling who drank, students who indicated not having a parent who drank were less likely (CI = .32–.59; *p* < .001) to report drinking, while those who reported not having a sibling who drank were less likely (CI = .36–.67; *p* < .001) to report drinking. Again, similar to smoking, academic achievement was associated with alcohol use with participants with the lowest academic achievement having the highest odds of reporting past 30-day alcohol use.

## Discussion

This study explored how romantic aspiration network characteristics at the individual level (in-degree and out-degree) are associated with smoking and drinking among 10th-grade students in 4 high schools. The data show these students’ network characteristics, parental or sibling substance use and academic performance are significantly associated with their likelihood of reporting smoking or drinking in the past 30 days.

An increase in a student’s in-degree (i.e. the number of incoming romantic aspiration nominations) was associated with a higher likelihood of reporting alcohol use during the past 30 days. Although for our sample this association was moderate in size, the relationship is supported by available research, showing that adolescents who are more popular (in this case, more romantically desired) are likely to be more social and to engage in behaviours perceived as socially desirable by their peers. Studies have shown that leisure time activities of adolescents take place in settings outside the home, usually at parties and gatherings in which risky behaviours (such as smoking and drinking) occur (Engels & ter Bogt, 2001). With studies finding support for the social function of drinking, our finding supports the notion socially adjusted/popular students were more likely to drink compared to their counterparts. As a result, the adolescents who are the most romantically desired are more likely to drink either because they frequent social gatherings which increase alcohol availability or because being ‘romantically desired’/ popular provides them a social status that encourages drinking.

We found that with an increase in out-degree (number of romantic nominations made), adolescents were less likely to report 30-day tobacco use. This finding suggests that adolescents actively seeking or aspiring to date are less likely to report smoking – an argument supported by Ennett and Bauman (1993) who hypothesised that smoking causes social isolation; hence, adolescents who aspire to develop intimate relations are more likely to abstain from smoking. In addition, Hall and Valente (2007) in their study of adolescent friendship networks and their influence on smoking suggested that adolescents who are non-smokers were unlikely to reciprocate friendships initiated by smoking. Thus, adolescents seeking to be sociable and romantically desired are less likely to report smoking because it could result in being socially undesirable.

Our results also reinforce prior studies’ findings regarding the association between familial substance use behaviours and adolescent substance use. We found that sibling smoking, and not parent smoking, was associated with adolescent smoking (Avenevoli & Merikangas, 2003; Vink, Willemsen, & Boomsma, 2003). This finding supports the existing literature reporting that siblings do matter, especially regarding transmission of influence among adolescents within a family unit (Kothari, Sorenson, Bank, & Snyder, 2014). The lack of association, in our samples, between parental smoking behaviour and adolescents’ report of smoking could be a result of the indirect effect (an effect possibly mediated by peers – i.e. parental smoking influenced associations with smoking peers) of parental smoking behaviour on adolescents’ smoking (Fergusson, Lynskey, & Horwood, 1995; O’Loughlin, Paradis, Renaud, & Gomez, 1998).

Consistent with prior studies, both parental and sibling drinking was associated with adolescent drinking within our sample (Avenevoli & Merikangas, 2003; Vink et al., 2003). This finding corroborates existing studies, showing there is a strong association between siblings’ and parents’ drinking patterns and the drinking patterns of adolescents in the home (Cohen & Rice, 1997; McGue, Sharma, & Benson, 1996; Windle, 2000). One of the mechanisms proposed to explain this association is that siblings and parents who consume alcohol model this behaviour directly to adolescents and are consequently the influential precursors of adolescent alcohol use within a family system (Kothari et al., 2014; van der Vorst, Engels, Meeus, Deković, & Van Leeuwe, 2005).

Despite the contributions this study makes to the literature, important limitations are worthy of notice. The main limitation of this study is its cross-sectional design which does not allow establishing causal relationships. Another issue which might also be considered a limitation is that the romantic network employed in the study was aspirational and not actual/existing romantic relationships. However, given that most romantic relations begin as an aspiration/attraction, this study is unique in that it examines this particular stage of budding romantic ties and its associations with substance use.

Notwithstanding these limitations, this study provides evidence that adolescent romantic aspirations/attractions can help researchers and practitioners better understand adolescents’ substance use behaviour. This finding is important and has significant implications for future research and prevention efforts: first, even though other network types (such as friendship networks) may impact substance use behaviour, romantic relations/aspirations are different and might have a stronger impact on adolescents’ substance use initiation due to the intensity of the relationship and the desire to please or be acceptable to the other person (Booth, Marsiglia, Nuňo-Gutiérrez, & Perez, 2014; Collins, 2003). Researchers would do well, in the future, to compare different types of networks to better understand their impact. Second, the implication this finding has for further research is that romantic aspirations/relations could serve as bridge between users and non-users; hence, researchers need to pay attention to how romantic associations (and not just friendship associations) might be a conduit for influence transmission. Given that there are very few studies examining adolescents’ romantic network characteristics and their influence on substance use, these findings suggest a need for further empirical studies related to adolescents’ social networks.

## Figures and Tables

**Table 1.** Characteristics of students in four schools in the Los Angeles area, participating in the Social Networks Study.

	School 1 (%) *n* = 376	School 2 (%) *n* = 276	School 3 (%) *n* = 204	School 4 (%) *n* = 254
*Age (Mean, SD)*	15.6 (.63)	15.6 (.59)	15.5 (.56)	15.6 (.53)
*Gender*				
Female	51.1	55.1	51	52
*Ethnicity*				
Hispanic/Latino	54	53.1	67.2	59.4
*Academic achievement*				
Mostly A’s and B’s	36.9	40.6	26.6	32.1
Mostly B’s and C’s	28.5	29	34.4	35.4
Mostly C’s and D’s	23.7	20.1	28.7	21.2
Mostly D’s and F’s	10.2	10.5	10.2	11.3
*Socio-economic status*				
Qualify for reduced lunch	79.7	93.4	95.4	88.0
# of rooms/people in the household (Mean, SD)	1.1(.74)	.82 (.54)	.83 (.63)	.85 (.47)
*Have parents who drink alcohol*				
Yes	45.9	49	59.5	57.1
No	54.1	51	4.05	42.9
*Have siblings who drink alcohol*				
Yes	67.1	36	36.4	36.2
No	32.9	64	63.6	63.8
*Have parents who smoke*				
Yes	24.9	28.8	35.4	26.1
No	75.1	71.2	64.6	73.9
*Have siblings who smoke*				
Yes	15.1	14.5	19.6	18.3
No	84.9	85.5	80.4	81.7
*Substance use (10th Grade)*				
Alcohol	28.9	35.6	39.5	40.6
Tobacco	8.1	11.9	8.6	9.4
*Out-degree (Mean, SD)*				
Romantic network	1.18 (1.81)	.95 (1.62)	1.27 (1.72)	.76 (1.51)
*In-degree (Mean, SD)*				
Romantic network	1.18 (1.44)	.95 (1.1)	1.27(1.09)	.76 (1.14)

**Table 2.** Logistic regression analysis showing association between intrapersonal, interpersonal and romantic network characteristics and adolescents’ 30-day tobacco use.

						95% C.I.for OR	
						
	*B*	S.E.	Wald’s *χ*^2^	OR	*p*	Lower	Upper
Age	.749	.225	11.099	2.114	.001	1.361	3.284
Gender	−.498	.257	3.759	.608	.053	.367	1.005
Ethnicity(1)	−.076	.258	.087	.927	.768	.559	1.536
Academic Achievement			16.801		.002		
Mostly D’s and F’s	1.503	.392	14.722	4.496	.000	2.086	9.689
Mostly C’s and D’s	.975	.375	6.751	2.652	.009	1.271	5.535
Mostly B’s and C’s	.497	.348	2.047	1.645	.152	.832	3.251
Mostly A’s and B’s (ref)							
Reduced lunch (No)	−.055	.411	.018	.947	.894	.423	2.119
Parent smoke (No)	−.016	.275	.003	.985	.955	.574	1.688
Sibling smoke (No)	1.334	.275	23.525	3.797	.000	2.215	6.510
In-degree	.020	.126	.025	1.020	.875	.797	1.306
out-degree	−.194	.092	4.457	.824	.035	.688	.986
School 1	−.435	.369	1.387	.647	.239	.314	1.335
School 2	.419	.340	1.515	1.520	.218	.781	2.959
School 3	−.064	.385	.028	.938	.868	.441	1.996
School 4 (ref)							
Constant	−6.437	1.135	32.141	.002	.000		
Test		*χ^2^*	*df*	*p*			
Overall model evaluation							
*R*^2^	.178						
*χ*^2^		75.758	15	.000			
Goodness of fit test							
*Hosmer & Lemeshow*		8.934	8	.348			

**Table 3.** Logistic regression analysis showing association between intrapersonal, interpersonal and network characteristics and adolescents’ 30-day alcohol use.

						95% C.I. for OR	
						
	*B*	S.E.	Wald’s *χ*^2^	OR	*p*	Lower	Upper
Age	.095	.133	.514	1.100	.473	.848	1.427
Gender	.221	.156	2.009	1.247	.156	.919	1.692
Ethnicity	−.040	.092	.185	.961	.667	.802	1.151
Academic Achievement			53.106		.000		
Mostly D’s and F’s	1.658	.267	38.579	5.249	.000	3.111	8.858
Mostly C’s and D’s	1.245	.224	30.927	3.473	.000	2.240	5.387
Mostly B’s and C’s	.642	.184	12.128	1.900	.000	1.324	2.726
Mostly A’s and B’s (ref) Reduced lunch (No)	.135	.243	.307	1.144	.580	.710	1.843
Parent alcohol (No)	−.831	.156	28.345	.435	.000	.321	.591
Sibling alcohol (No)	−.705	.156	20.543	.494	.000	.364	.670
In-degree	.171	.069	6.185	1.186	.013	1.037	1.357
Out-degree	.065	.046	1.970	1.067	.160	.975	1.168
School 1	−.685	.215	10.180	.504	.001	.331	.768
School 2	−.121	.211	.330	.886	.566	.585	1.340
School 3	−.090	.223	.163	.914	.686	.591	1.414
School 4 (ref)							
Constant	−.906	.663	1.867	.404	.172		
Test			*χ^2^*	*df*	*p*		
Overall model evaluation							
*R*^2^	.211						
*χ*^2^			152.983	15	.000		
Goodness of fit test							
*Hosmer & Lemeshow*			3.172	8	.923		
